# Revealing Pathological Auditory Central Inhibition in Tinnitus Using Cortical Auditory Evoked Potentials Responses to Contralateral Acoustic Stimulation

**DOI:** 10.1002/brb3.71007

**Published:** 2025-10-29

**Authors:** Zhou Qian, Wang Qixuan, Jiang Wenling, Wang Yiting, Li Haifeng, Huang Meiping, Yang Lu, Ren Yan, Sheng haibin, Li Bei, Huang Zhiwu

**Affiliations:** ^1^ Department of Otolaryngology‐Head and Neck Surgery Shanghai Ninth People's Hospital, Shanghai Jiao Tong University School of Medicine Shanghai China; ^2^ Faculty of Hearing and Speech Science, College of Health Science and Technology Shanghai Jiao Tong University School of Medicine Shanghai China; ^3^ Shanghai Key Laboratory of Translational Medicine on Ear and Nose Diseases Shanghai China; ^4^ Ear Institute Shanghai Jiao Tong University School of Medicine Shanghai China; ^5^ Department of Facial Plastic and Reconstructive Surgery Eye & ENT Hospital of Fudan University Shanghai China; ^6^ ENT Institute Eye & ENT Hospital of Fudan University Shanghai China; ^7^ NHC Key Laboratory of Hearing Medicine Fudan University Shanghai China; ^8^ Department of Otolaryngology‐Head and Neck Surgery Shanghai Renji Hospital, Shanghai Jiao Tong University School of Medicine Shanghai China

**Keywords:** auditory central inhibition, cortical auditory evoked potentials, contralateral suppression, tinnitus, tinnitus masking therapy

## Abstract

**Objective:**

To test the hypothesis that auditory central inhibition is reduced in tinnitus patients and explore whether improving this inhibitory function could alleviate tinnitus severity.

**Methods:**

We recruited 16 chronic tinnitus patients and 14 age‐matched healthy controls, all of whom exhibited clinically normal audiometric thresholds. Electroacoustic measures, cortical auditory evoked potentials (CAEP), were used to assess neural activity. Contralateral noise stimulation was employed to evaluate contralateral suppression (CS) of CAEP amplitude.

**Results:**

Significant differences in CS of N100 and P300 amplitude were observed between tinnitus patients and healthy controls, indicating impaired central inhibition in tinnitus. After 3 months of tinnitus masking therapy, patients showed significant improvements in CS of N100 and P300 amplitudes, which correlated with a reduction in tinnitus handicap inventory scores. Receiver operating characteristic curve analysis confirmed that changes in CS, especially in the P300 amplitude, reliably reflect treatment efficacy.

**Conclusions:**

This study highlights the critical role of central auditory inhibition in tinnitus pathophysiology and suggests that neurophysiological markers, particularly changes in CS of P300 amplitude, could serve as reliable biomarkers for evaluating treatment outcomes.

**Significance:**

These findings pave the way for developing targeted therapies aimed at restoring central auditory inhibition, offering more effective and personalized treatment strategies for tinnitus patients.

## Graphical abstract

Chronic tinnitus shows weakened contralateral suppression of cortical auditory evoked potentials, pointing to deficient central inhibition. Personalized masking therapy partially restores this inhibition, and the recovery of contralateral‐suppressed P300 amplitude closely tracks symptom relief—highlighting P300 CS as a biomarker for diagnosis and treatment monitoring.

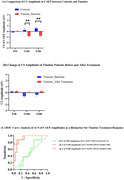

**Abbreviations**
△CS: change in CS
AUC: area under the curve
CAEP: cortical auditory evoked potential
Controls: control group
CS: contralateral suppression
EEG: electroencephalography
EG: effective group
HL: hearing loss
ICA: independent component analysis
IG: ineffective group
MML: minimum masking level
MOC: medial olivocochlear
MRI: magnetic resonance imaging
NH: normal hearing
OAEs: otoacoustic emissions
RI: residual inhibition
ROC: receiver operating characteristic
SFRs: spontaneous firing rates
TEOAE: transient evoked otoacoustic emissions
THI: tinnitus handicap inventory
TS: tinnitus group
VAS: visual analog scale

## 1 Introduction

Tinnitus is a persistent auditory perception in the absence of external sound stimuli, affecting approximately 10%–15% of the global population (Bauer 2018). Among those affected, about 20% experience significant impairments in quality of life, often accompanied by emotional distress and sleep disturbances (Pattyn et al. 2016). Despite its high prevalence, there are currently no effective pharmacological or surgical treatments for tinnitus. Sound‐based interventions—particularly sound masking therapy—have been widely adopted as safe and convenient options; sustained therapy can reduce perceived loudness and symptom burden (Hobson et al. 2012; Neff et al. 2019; Schad et al. 2018). Recent longitudinal imaging demonstrates that sound therapy both recalibrates auditory‐network connectivity—enhancing auditory‐limbic coupling and normalizing thalamo‐auditory links—and reverses structural abnormalities in the right middle‐frontal/pre‐central cortex and bilateral middle cerebellar peduncles (Chen et al. 2021; Lv et al. 2021).

Tinnitus likely reflects an excitation–inhibition (E/I) imbalance in the auditory system driven by peripheral damage and maladaptive central plasticity (Henry 2014; Langguth 2024; Sedley 2019). Evidence for hyperexcitability includes elevated spontaneous firing and neural synchrony in the dorsal cochlear nucleus, inferior colliculus, and auditory cortex (Berger and Coomber 2015; Gao et al. 2016; Zhang et al. 2016). Growing data also implicate reduced inhibition—for example, diminished GABAergic activity and impaired potassium‐channel function—yet its role in pathogenesis and treatment remains underexplored (Pilati et al. 2012).

Auditory inhibition involves a descending corticofugal system, which projects from the auditory cortex to subcortical nuclei and the cochlea (Ryugo 2011). Efferent control of cochlear gain via the medial olivocochlear (MOC) pathway can be indexed peripherally by contralateral suppression (CS) of otoacoustic emissions (Keppler et al. 2010; Lalaki et al. 2011). However, whether contralateral noise also suppresses cortical auditory responses—measured as CS of cortical auditory evoked potentials (CAEP)—and whether such CS‐CAEP indexes central inhibitory control in tinnitus remain open questions.

Previous studies have explored CAEP‐based markers of excitation‐inhibition imbalance in tinnitus. For example, Morse et al. reported altered onset‐offset cortical responses and changes in P200 amplitude in tinnitus patients, suggesting impaired inhibitory processing (Morse and Vander Werff 2023). Additionally, Julia Campbell and colleagues have extensively investigated auditory sensory gating—particularly paired‐click CAEP paradigms—as a measure of cortical inhibition. Their work demonstrates that individuals with tinnitus show impaired gating (i.e., reduced suppression of the second auditory response), especially in the P1–P2 components, even in the absence of audiometric hearing loss (Campbell et al. 2018). Notably, these gating deficits correlate with tinnitus severity and involve altered cortical source activation, including reduced frontal lobe engagement and compensatory parietal recruitment (Campbell et al. 2018; Campbell et al. 2019; Ralston et al. 2024). These findings support the role of central inhibitory dysfunction in tinnitus and highlight auditory gating as a potential biomarker. However, while sensory gating paradigms index inhibition across repeated stimuli, direct evidence of CS effects within CAEP—analogous to the well‐established otoacoustic emissions (OAE) suppression paradigm—remains limited (Jedrzejczak et al. 2022; Morlet et al. 2019; Rao et al. 2020). Whether CS‐CAEP reflects central auditory inhibition and can serve as a reliable neurophysiological biomarker has not yet been established. Given the growing interest in cortical inhibition and its therapeutic relevance, further investigation of CS effects in CAEP may help clarify their diagnostic and prognostic utility in tinnitus.

Based on these insights, we hypothesize that tinnitus is associated with reduced central auditory inhibition and that sound masking therapy may alleviate symptoms by partially restoring this inhibitory function. To probe this mechanism, we propose a novel approach to assess cortical inhibition by measuring CS effects during CAEP recording. Specifically, we set out to test whether contralateral noise suppresses cortical responses in normal‐hearing tinnitus patients as it does in controls, and whether any such inhibitory effect changes after masking therapy. We further predict that CS of the N100 and P300 amplitudes will be attenuated in tinnitus versus controls, and that within‐patient increases in CS from pre‐ to post‐therapy will track clinical improvement. By linking neurophysiological markers to clinical outcomes, this work aims to establish CS of CAEP as a potential biomarker for both diagnosis and treatment response, advancing the development of personalized, mechanism‐based interventions for chronic tinnitus.

## 2 Methods

### 2.1 Data Sources, Study Population, and Ethics

We recruited 16 tinnitus patients (Tinnitus, nine males; mean age: 33.9 ± 11.9 years) and 14 healthy controls (controls, seven males; mean age: 28.6 ± 5.6 years) from the Hearing Center of Shanghai Ninth People's Hospital between September 2022 and March 2024. The age and sex distributions between the two groups did not differ significantly in statistical terms. Tinnitus patients completed a questionnaire covering demographic details, health status, and medication use. Tinnitus patients provided information on the duration, characteristics, and laterality of their tinnitus. Tinnitus patients underwent standard audiometric evaluations performed by an otolaryngologist. Inclusion criteria included being 18 years or older, experiencing persistent tinnitus for over 3 months, being able to participate in the study, and having no structural brain abnormalities or profound hearing loss in the affected ear. To target central inhibitory mechanisms while minimizing confounding from peripheral impairment, enrollment was restricted to tinnitus participants with normal audiometric thresholds. Exclusion criteria included pulsatile tinnitus, Meniere's disease, otosclerosis, sudden deafness, contraindications to MRI, or a history of brain diseases such as stroke or head injury confirmed by conventional MRI. Participants with central nervous system disorders, including neurological conditions such as stroke, multiple sclerosis, epilepsy, Parkinson's disease, or cognitive disorders such as dementia, were excluded from the study to prevent confounding effects on the neurophysiological measures. This investigation was conceived as a mechanism‐focused exploratory study conducted under a fixed recruitment window and stringent eligibility criteria; accordingly, no a priori power analysis was undertaken. All participants provided written informed consent, and this study was approved by the Ethics Committee of the Shanghai Ninth People's Hospital, located in Shanghai, China (Approval No.: SH9H‐2022‐T379‐1).

Tinnitus patients were assigned to receive tinnitus masking therapy as part of the intervention, while healthy controls served as a non‐intervention reference group. This design ensured a clear comparison between the treatment effects and baseline auditory and psychological conditions. Individualized sound masking therapy was administered three times daily for 45 min per session over a 3‐month period. The masking sounds were customized based on the individual's tinnitus frequency characteristics, using narrowband noise tailored to effectively cover the perceived tinnitus. The therapeutic sound was recorded using a clinical audiometer and delivered to the patients’ mobile device or similar platform, to be listened to through headphones. During each session, patients were instructed to relax and shift their attention away from the tinnitus. They were advised to adjust the sound volume to a comfortable level that sufficiently masked the tinnitus without causing listening fatigue or discomfort. Patients were encouraged to sit or lie down in a relaxed position and avoid distracting activities such as reading, using electronic devices, or watching television. Importantly, patients were not required to focus on the tinnitus or the masking sound. Instead, they were encouraged to let their thoughts flow freely, fostering a relaxed and pressure‐free mental state. This environment was designed to help reduce the emotional and cognitive burden associated with tinnitus, potentially improving treatment outcomes.

Tinnitus severity was evaluated using the tinnitus handicap inventory (THI) before the first treatment and after the final session (Newman et al. 1996). The change in THI (ΔTHI) for each patient was calculated as follows: ΔTHI = baseline THI − post‐treatment THI. Tinnitus loudness was quantified using the visual analog scale (VAS, 0–10 scale, higher scores indicating greater loudness), and the change in VAS (ΔVAS) for each patient was calculated as follows: ΔVAS = baseline VAS − post‐treatment VAS. Significant effectiveness was defined as a minimum improvement of seven points in the overall THI score after the sound masking intervention (Cai et al. 2017; Zeman et al. 2011). Based on this criterion, patients were categorized into an effective group (EG) and an ineffective group (IG).

### 2.2 Audiometric Tests

All participants completed an audiological evaluation performed by a trained audiologist, conducted at the Hearing Center of Shanghai Ninth People's Hospital. Pure tone audiometry was performed across octave intervals from 0.25 to 8 kHz, including frequencies of 3 and 6 kHz. Tinnitus frequency and loudness were assessed using standard audiometric methods, where participants selected pure tones that best matched their perceived tinnitus. The most frequently matched frequency was identified as the tinnitus frequency, with loudness expressed in dB SL above the hearing threshold.

Minimum masking level (MML) was defined as the lowest level of narrowband noise required to render the tinnitus inaudible. We used narrowband noise centered on the tinnitus frequency as the stimulus. For participants with unilateral tinnitus, testing was conducted on the affected ear; for those with bilateral tinnitus, both ears were tested. The stimuli began at low levels and were increased in 2 dB increments until the tinnitus was completely masked. This process was repeated 2–3 times, and the average result was recorded if variations were within 5 dB.

Residual inhibition (RI) refers to the short‐term suppression of tinnitus following sound stimulation. After the masker was turned off, tinnitus may return to its baseline level or diminish temporarily before resuming. We tested RI at an intensity level of 10 dB above the MML, presenting a 60‐second narrowband noise stimulus centered on the tinnitus frequency. After each RI trial, the participant was asked whether their tinnitus perception had disappeared, decreased, remained the same, or increased. Complete disappearance or reduction of tinnitus more than a few seconds following the termination of the narrowband noise was classified as RI (+), while no change or a perceived increase in tinnitus loudness was classified as RI (−). No participants reported worsening of tinnitus following stimulation.

### 2.3 Transient Evoked Otoacoustic Emissions (TEOAE) Recordings

TEOAEs provide frequency‐specific information about cochlear function and outer hair cell motility. We assessed CS of TEOAEs to evaluate the MOC efferent reflex (Stamper and Johnson 2015). TEOAEs were elicited using 60 dB peak equivalent SPL (peSPL) linear click stimuli at a rate of 19.3/s, both with and without a 50 dB SL contralateral white noise suppressor, without removing the probe. The suppressor intensity was kept below the stapedius reflex threshold for all participants. Responses were averaged over 2080 sweeps to ensure at least 70% stimulus stability. Amplitudes of TEOAEs were measured with and without contralateral noise stimuli. CS of TEOAE was defined as the difference in dB between two TEOAE recordings, calculated by subtracting TEOAE amplitudes, with contralateral noise stimulation from TEOAE amplitude in the absence of sound at half octave frequency bands centered around frequencies 1, 2, 3, 4, and 5 kHz, as well as across frequencies.

### 2.4 Electrophysiological Measure

Continuous electroencephalography (EEG) data were collected at a 1000 Hz sampling rate using a 256‐channel EEG system (HydroCel Geodesic Sensor Net, EGI). Participants were instructed to remain still, awake, and focused on a point in front of them while seated in a soundproof room. The impedance of all electrodes was kept below 50 kΩ, and CAEP was recorded with an online reference at Cz.

The EEG recording included two auditory tasks presented via Psychtoolbox. In the first task, participants completed an auditory oddball paradigm under a no‐noise condition. In this task, two types of stimuli—frequent (standard) and rare (target)—were presented in random order. Participants were instructed to press a button with one hand whenever they detected a rare target. The oddball task consisted of 50–53 target stimuli (20%) and 200–205 standard stimuli (80%), with the first five stimuli being standards. At least two standard stimuli were presented between each target stimulus. In the no‐noise condition, stimuli were presented monaurally, with the left and right ears tested in randomized order.

In the second task (noise condition), participants completed an auditory oddball task in which the same auditory stimuli were presented monaurally to one ear, randomly assigned, while continuous narrowband noise—matched to the target frequency—was simultaneously delivered to the contralateral ear. The noise intensity was set at 40 dB above each participant's hearing threshold at the corresponding center frequency. Both ears were tested in random order, with each ear serving as the stimulus ear while the contralateral ear received noise in separate trials. A 1‐min break was given between conditions to minimize fatigue.

For both tasks, the target stimuli had a frequency of 4 kHz, corresponding to the typical tinnitus frequency in patients, while standard stimuli were presented at 1 kHz. Each stimulus lasted 200 ms with 10 ms rise and fall times, and the inter‐stimulus interval was 800 ms. While experimenters could not be fully blinded to the noise‐on/noise‐off condition during acquisition, we minimized bias using standardized task scripts, automated preprocessing, and artifact rejection. Task order and contralateral‐noise side were randomized/counterbalanced across participants. Auditory stimuli were delivered through insert earphones (EARTONE 3A), with the intensity calibrated to 65 dB SPL. The sound level meter was used to ensure accurate calibration. Participants maintained their gaze on a fixed cross throughout the tasks to reduce distractions from visual attention shifts.

### 2.5 CAEP Data Analyses

CAEP data were processed offline using MATLAB R2022b and the EEGLAB toolbox (available at EEGLAB Toolbox). EEG signals were bandpass filtered between 1 and 30 Hz, and data were re‐referenced to an average reference. Independent component analysis (ICA) was applied to correct for EOG artifacts. The data were then segmented from 100 ms before stimulus presentation to 600 ms after stimulus onset. Epochs containing artifacts—defined as a maximum amplitude exceeding ±150 µV or a maximum voltage gradient of 50 µV/ms—were excluded.

For each participant, the average of all artifact‐free trials for each stimulus type (standard and target) was computed. Cz was chosen as the primary channel for presenting time‐domain data based on the observed peak morphology. The P50 amplitude was measured as the maximum voltage within the 20 to 120 ms post‐stimulus window, while the N100 amplitude was assessed as the minimum voltage within the 80 to 180 ms window. N100 amplitude was analyzed as the absolute value. The P300 amplitude was defined as the maximum voltage occurring between 250 and 500 ms in response to target stimuli. The accuracy of target detection during the P300 session was above 90% for all participants.

Due to the lack of a hypothesis regarding the P300 latency and considering the relatively long duration of the P300 response without significant latency differences, we did not analyze the P300 latency. The primary outcome measure was the P300 amplitude, with N100 and P50 amplitudes serving as secondary measures. Amplitudes of CAEP were measured with and without contralateral noise stimuli. CS of CAEP amplitude was defined as the difference in µV between two CAEP recordings, calculated by subtracting the CAEP amplitude with contralateral noise stimulation from the CAEP amplitude without noise.

### 2.6 Statistical Analysis

Continuous variables are presented as means (± standard deviation), while categorical variables are expressed as proportions. The Kolmogorov–Smirnov test was used to assess the normality of the data. For normally distributed data, intergroup differences were analyzed using independent samples *t*‐tests. For non‐normally distributed data, the Mann–Whitney *U* test was applied to evaluate differences in continuous variables between groups.

Demographic, psychological characteristics, and CAEP amplitudes were compared between the tinnitus group (tinnitus) and the control group (controls) using the two‐sample *t*‐test for continuous variables and the chi‐square test for categorical variables. P50, N100, and P300 amplitudes were analyzed using a two‐way repeated measures ANOVA, with “condition” (no‐noise and noise) as the within‐subject factor and “group” (tinnitus and controls) as the between‐subject factor. Bonferroni post‐hoc corrections were applied when appropriate.

For pre‐ and post‐treatment comparisons in the tinnitus group, the paired‐samples *t*‐test was used. Spearman correlation analysis was performed to explore potential relationships between clinical and electrophysiological variables. Receiver operating characteristic (ROC) curves were generated to assess diagnostic performance. Statistical significance was set at *p* < 0.05. All analyses were performed using SPSS statistical software (Version 23.0).

## 3 Results

### 3.1 Demographic and Clinical Traits

**FIGURE 1:**
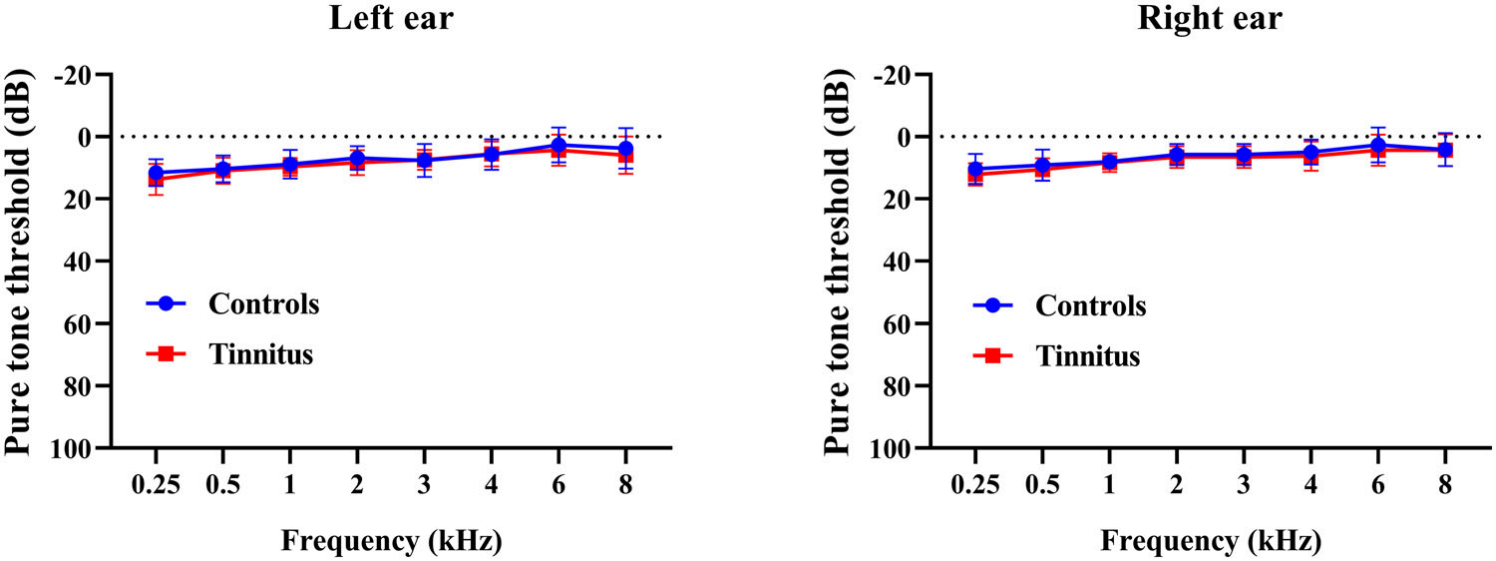
Pure tone audiometry of subject groups. Plots indicate group mean and 1 SD at each frequency/ear.

Table 1 shows the demographic and clinical characteristics of the participants. This study recruited two groups of participants: a chronic tinnitus group (tinnitus, *n* = 16) and a healthy control group (controls, *n* = 14). There were no significant differences in age or sex between the two groups (*p* > 0.05). All participants in the tinnitus and control group had hearing thresholds of ≤20 dB across frequencies from 0.5 to 8 kHz (see Figure 1).

**TABLE 1:** Demographic and clinical variables for tinnitus patients and controls.

Variables (unit)	Tinnitus group (*n* = 16) mean ± SD (or count)	Control group (*n* = 14) mean ± SD (or count)	*p*‐value
Age (*y*)	27.7±9.1	28.6±5.6	0.755
Sex (M/F)	9/7	7/7	0.732
Tinnitus characteristics			
Duration (*y*) Location (unilateral/bilateral) THI scores VAS scores Tinnitus match CF (Hz) RI (+)/RI (−)	2.4±1.9 7/9 31.6±18.3 4.61±.5 4532±2044 8/8	/ / / / / /	
CS of average TEOAE amplitude (dB)	1.72±0.20	2.48±0.19	0.008*
CAEP amplitude (µV)			
P50 amplitude	0.42±0.13	0.52±0.13	0.495
N100 amplitude	1.07±0.17	1.78±0.22	0.012*
P300 amplitude	1.42±0.19	2.35±0.21	0.002*
CS of P50 amplitude	0.05±0.17	−0.14±0.13	0.099
CS of N100 amplitude	−0.86±0.23	0.26±0.26	0.002*
CS of P300 amplitude	−0.79±0.24	0.55±0.19	0.001*

*Note*: Analyses were conducted using independent‐samples *t* test, *p < 0.05.

Abbreviations: CAEP, cortical auditory evoked potentials; CF, center frequency; CS, contralateral suppression; F, female; HL, hearing loss; M, male; NH, normal hearing; RI, residual inhibition; SEM, standard deviation; THI, tinnitus handicap inventory; VAS, visual analog scale.

### 3.2 Comparison of TEOAE Results Between Tinnitus and Controls

**FIGURE 2:**
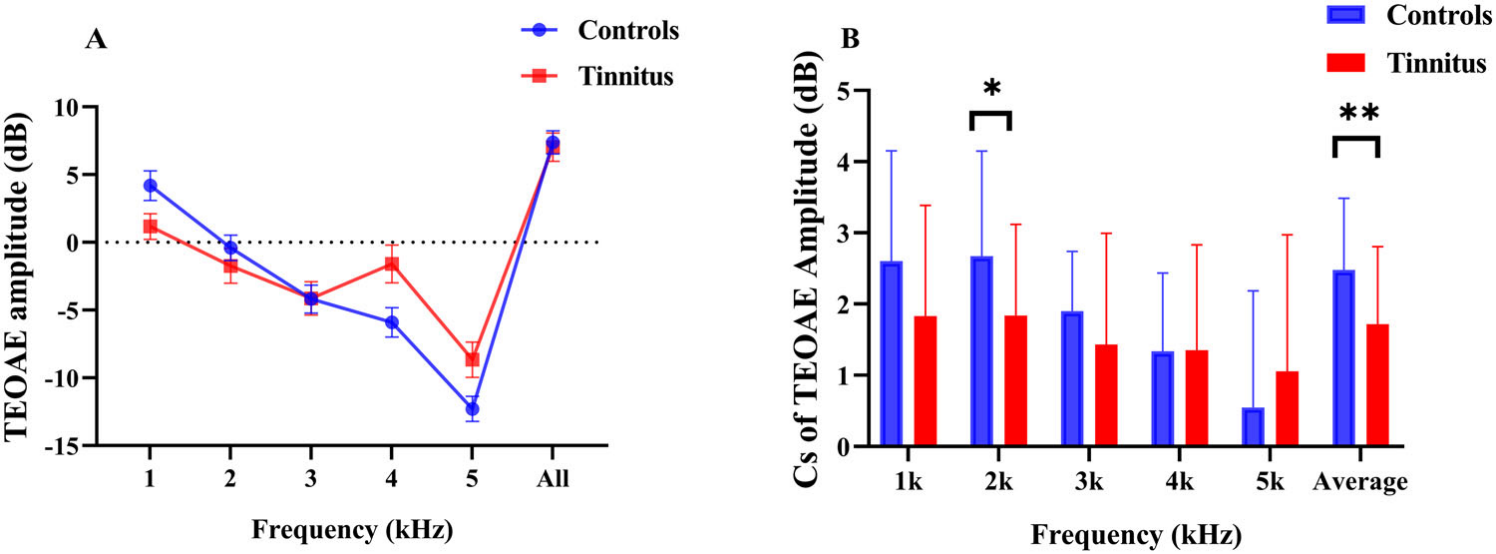
Group comparison of (A) TEOAE amplitude and (B) CS of TEOAE amplitude recorded from controls and tinnitus. CS of TEOAE amplitude was defined as the difference in dB between two TEOAE recordings. Analyses were performed by the two‐sample *t* test. *p < 0.05, **p < 0.01.

TEOAE amplitudes across frequencies did not differ significantly between the two groups (all *p* > 0.05; see Figure 2A). However, significant differences were observed in CS of TEOAE amplitude at 2 kHz and in the average values between Controls and Tinnitus (*t*(28) = 2.440, *p* = 0.018; *t*(28) = 2.723, *p* = 0.008, respectively; see Figure 2B).

### 3.3 Comparison of CAEP Results Between Tinnitus and Controls

**FIGURE 3:**
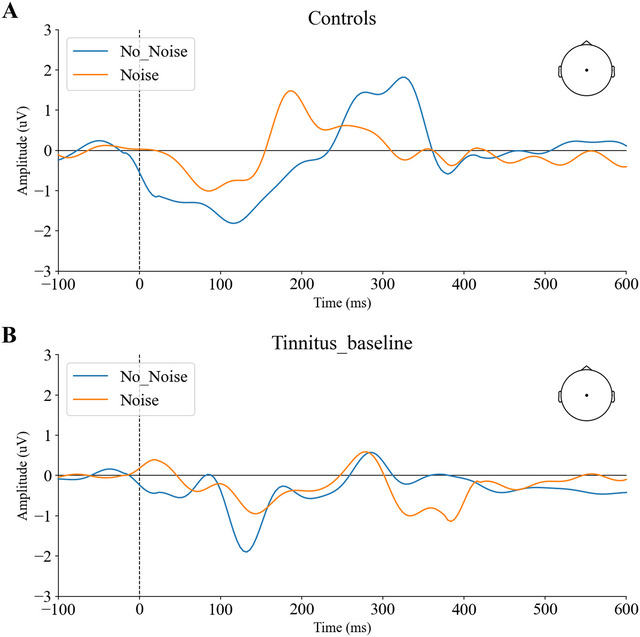
Evoked waveforms from the Cz electrode for the target stimulus were recorded under no‐noise (blue line) and noise (red line) conditions for controls (A)and tinnitus at baseline (B).

Figure 3A,B displays the grand average of the CAEP components recorded from the tinnitus and controls group, averaged across both left and right ears under no‐noise and noise conditions. We observed no significant differences in the amplitudes of P50, N100, and P300 between the left and right ears across all participants (*p* > 0.05). Consequently, subsequent analyses did not differentiate between the ears and combined data from both sides for evaluation.

**FIGURE 4:**
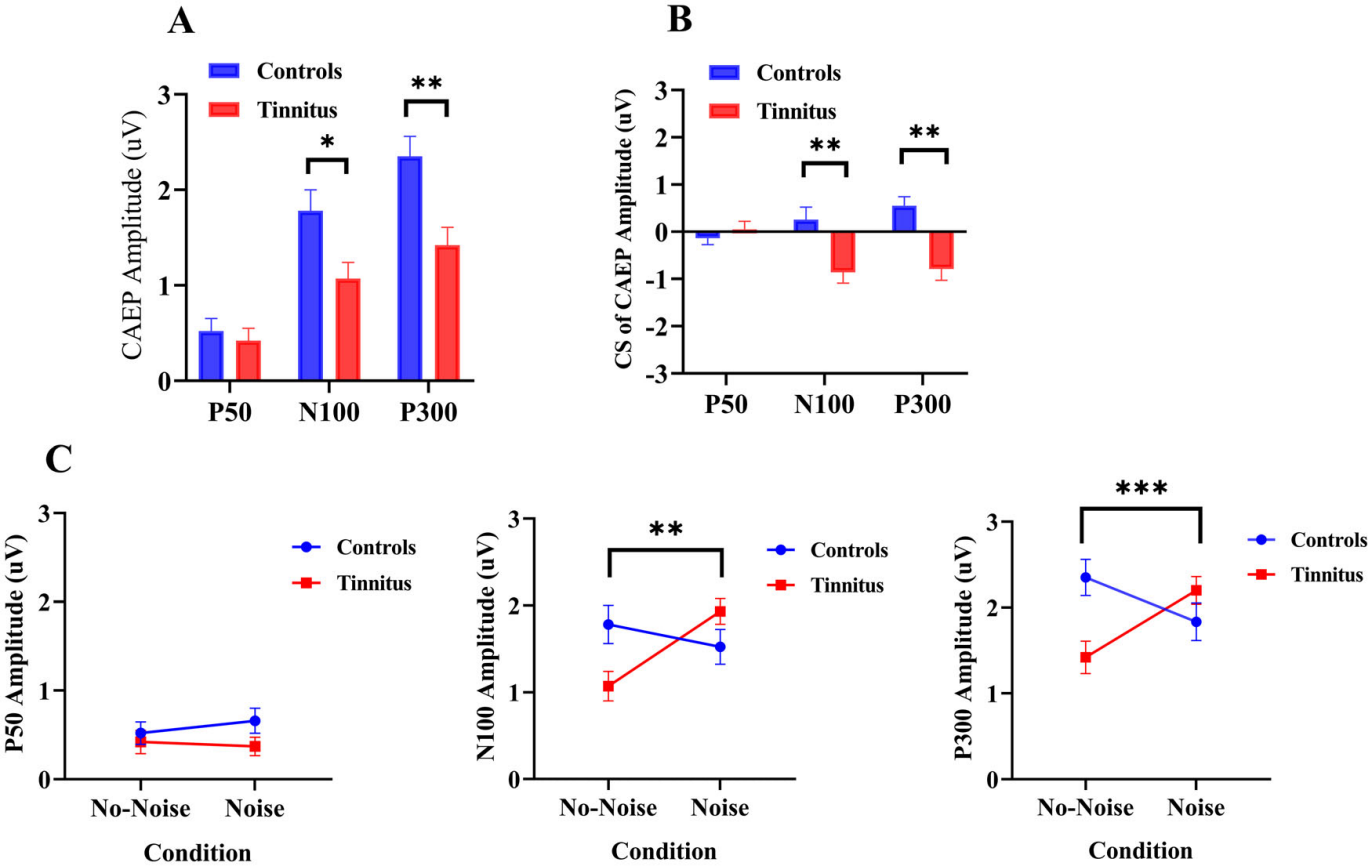
(A) Comparison of CAEP amplitude between controls and tinnitus under no‐noise conditions; (B) Comparison of CS of CAEP amplitude between controls and tinnitus; (C) amplitudes of P50, N100, and P300 components in tinnitus and controls under no‐noise and noise conditions. CS of CAEP amplitude was defined as the difference in µV between two CAEP recordings. *Note*: N100 amplitudes are plotted by their absolute value (the N100 peak is negative in voltage, but its magnitude is shown here as a positive for ease of comparison). *p < 0.05, **p < 0.01, ***p < 0.001.

For the P50 evoked by target stimuli, results indicated no significant differences in amplitude between the controls and tinnitus group under no‐noise conditions (*p* > 0.05). However, the mean N100 (a negative‐going deflection, plotted as absolute amplitude) and P300 amplitude were significantly lower in tinnitus patients than controls (*t*(28) = −2.589, *p* = 0.012; *t*(28) = 3.323, *p* = 0.002, respectively; see Figure 4A).

CS of CAEP amplitude was significantly lower for N100 amplitude and P300 amplitude in tinnitus compared to controls (*t*(28) = 3.208, *p* = 0.002; *t*(28) = 4.303, *p* < 0.001, respectively). There were no significant differences in CS of P50 amplitude between groups (*p* > 0.05) (see Figure 4B)

A two‐way repeated measures ANOVA revealed a significant interaction effect between “condition” and “group” for the P300 amplitude (*F*(1,28) = 18.545, *p* < 0.001). After Bonferroni correction, significant differences were observed only in controls (*p* = 0.018), with P300 amplitude lower in the noise condition compared to the no‐noise condition. A significant interaction effect for N100 amplitude was also found (*F*(1,28) = 10.294, *p* = 0.002), with the N100 amplitude higher in the Noise condition compared to the no‐noise for tinnitus (*p* = 0.001). No significant effects were found for P50 amplitude (*p* >0.05; see Figure 4C).

#### 3.3.1 Utility of CS as a Diagnostic Marker

**FIGURE 5:**
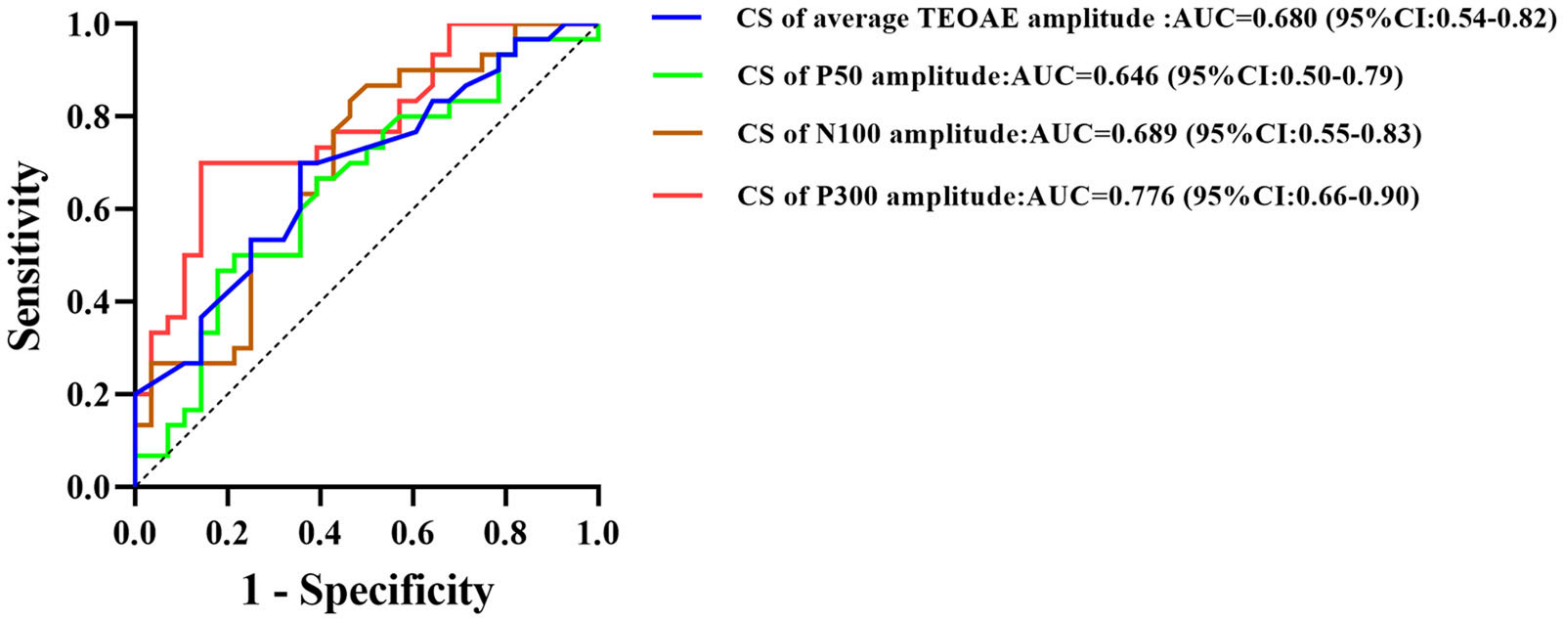
ROC curve distinguishing tinnitus from controls using CS metrics. Curve‐predictor mapping: solid red—CS of P300 amplitude; solid brown—CS of N100 amplitude; solid green—CS of P50 amplitude; solid blue—CS of average TEOAE amplitude. The dashed gray diagonal denotes chance performance. Higher values indicate better discrimination. Abbreviations: CS, contralateral suppression; TEOAE, transient‐evoked otoacoustic emission; ROC, receiver operating characteristic.

We evaluated whether CS of TEOAE amplitude or CAEP amplitude could serve as a biomarker for tinnitus. The ROC curve analysis revealed that the CS of P300 amplitude had the largest area under the curve (AUC) under the ROC curve (0.776, 95% CI: 0.656–0.895), indicating fair diagnostic accuracy (see Figure 5).

### 3.4 Comparison of Tinnitus Severity, TEOAE and CAEP Amplitude of Tinnitus Before and After Tinnitus Masking Treatment

**FIGURE 6:**
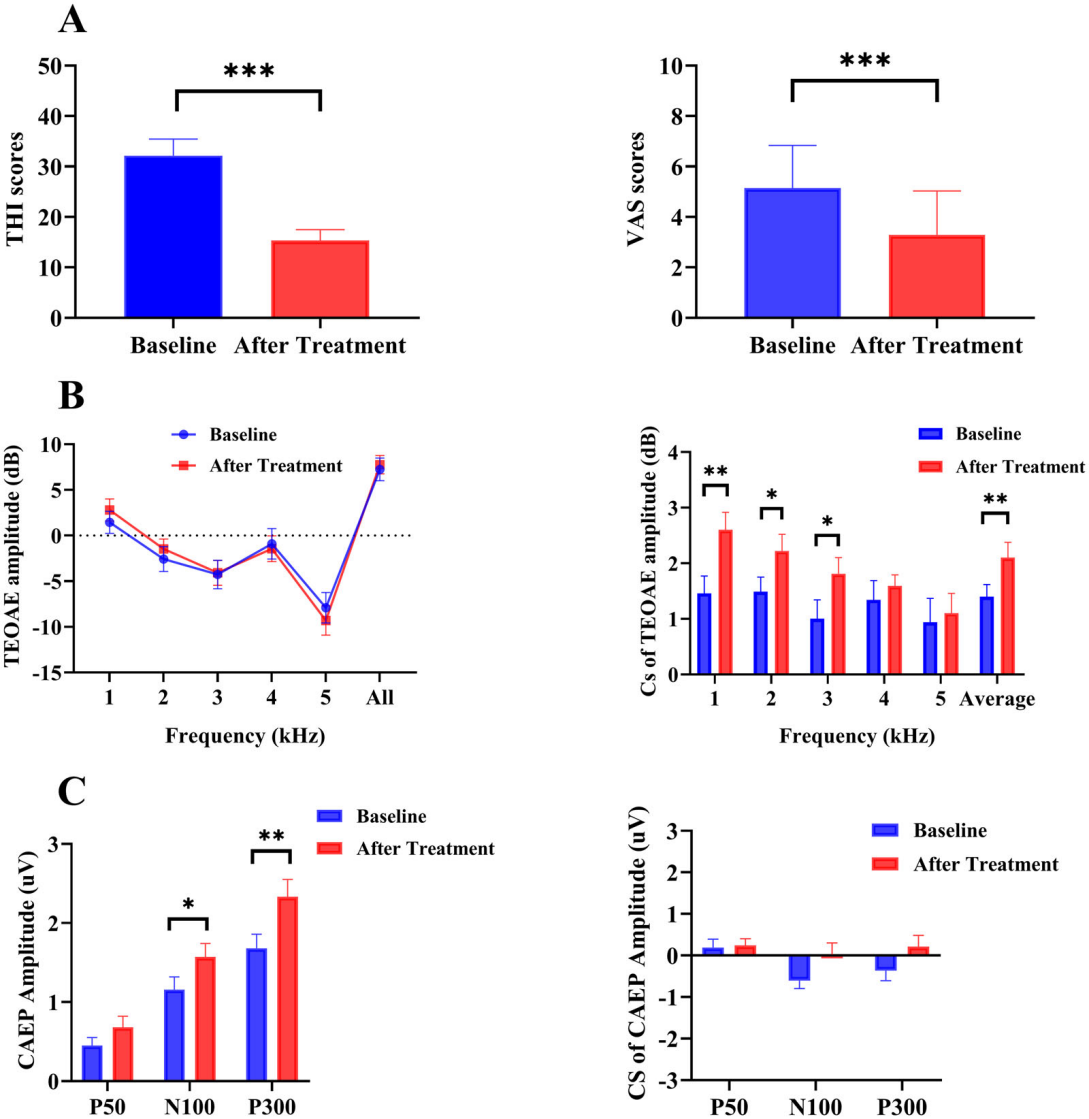
(A) Comparison of THI and VAS scores between baseline and post‐treatment for tinnitus. (B) Comparison of TEOAE amplitude and CS of TEOAE amplitude between baseline and post‐treatment for tinnitus. (C) Comparison of CAEP amplitudes and CS of CAEP amplitude between baseline and post‐treatment for tinnitus. *Note*: N100 amplitudes are plotted by their absolute value. *p < 0.05, **p < 0.01, ***p < 0.001.

Due to time and geographical constraints, only 12 of the 16 tinnitus participants returned for THI, VAS, TEOAE, and CAEP testing. Following treatment, significant reductions in tinnitus severity were observed, as indicated by the THI and VAS scores (*t*(11) = 5.143, *p* < 0.001; *t*(11) = 10.718, *p* < 0.001, respectively; see Figure 6A). While no significant changes were found in TEOAE amplitudes across frequency bands, the CS of TEOAE amplitudes at 1, 2, and 3 kHz, as well as the average value, showed significant improvement from baseline to post‐treatment (*p* < 0.05) (see Figure 6B).

Additionally, N100 and P300 amplitudes under the no‐noise condition were significantly enhanced post‐treatment (*t*(11) = 2.086, *p* = 0.048; *t*(11) = 3.988, *p* = 0.001, respectively). Although the CS of P50, N100, and P300 amplitudes were higher after treatment compared to baseline, these differences were not statistically significant (all *p* > 0.05) (see Figure 6C).

### 3.5 Comparison of Changes in TEOAE and CAEP Amplitude Between the Effective Group (EG) and Ineffective Groups (IG)

Among 12 tinnitus participants, eight were classified as responders (effective group, EG), defined by a reduction of seven points or more in their THI score, while four were classified as non‐responders (ineffective group, IG), with a reduction of less than seven points. The change in CS (ΔCS) of CAEP amplitude was calculated by subtracting the baseline CS of CAEP amplitude from the post‐treatment CS of CAEP amplitude. This same definition was applied to ΔCS of average TEOAE amplitude.

Table 2 shows that the ΔCS of average TEOAE amplitude, as well as the ΔCS of N100 and P300 amplitude, were significantly greater in the EG group compared to the IG group (*Z* = −2.068, *p* = 0.039; *Z* = −3.429, *p* = 0.001; *Z* = −3.613, *p* < 0.001, respectively; see Table 2). However, there were no significant differences between the EG and IG groups in terms of tinnitus duration (*p* > 0.05).

**TABLE 2:** Comparison of clinical variables, △CS of TEOAE and CAEP amplitude between the effective group (EG) and ineffective group (IG) in tinnitus patients.

Variables	EG groups (*n* = 8)	IG groups (*n* = 4)	*p*‐value
Duration of tinnitus (mean ± SD, *y*)	2.50±2.28	6.50 ±6.40	0.122
△CS of average TEOAE amplitude (mean ± SD, dB)	0.88±0.96	−0.18±0.25	0.039*
CAEP Amplitude (mean ± SD, µV)			
△CS of P50 amplitude △CS of N100 amplitude △CS of P300 amplitude	−0.05±0.38 1.58±0.41 1.40±0.39	−0.11±0.27 −1.24±0.24 −1.16±0.49	0.854 0.001* <0.001*

*Note*: Analyses were conducted using Mann–Whitney *U* test, *p < 0.05.

Abbreviation: △CS, change of contralateral suppression between the baseline and after treatment of tinnitus.

Spearman correlation analysis revealed significant positive correlations between ΔTHI and ΔCS of N100 amplitude (*r* = 0.638, *p* = 0.001) and ΔCS of P300 amplitude (*r* = 0.637, *p* = 0.001) (see Table 3). These findings suggest that improvements in the CS of N100 and P300 amplitude are associated with reductions in tinnitus severity. However, no significant correlation was observed between ΔVAS and the ΔCS of TEOAE or CAEP amplitude (*p* > 0.05; see Table 3).

**TABLE 3:** Correlation between changes in CS of TEOAE and CAEP amplitude and clinical traits.

	△THI	△VAS
△CS of average TEOAE amplitude	*r* = 0.316 *p* = 0.233	*r* = −0.351 *p* = 0.183
△CS of P50 amplitude △CS of N100 amplitude △CS of P300 amplitude	*r* = −0.238 *p* = 0.263 *r* = 0.638 *p* = 0.001* *r* = 0.637 *p* = 0.001*	*r* = −0.402 *p* = 0.052 *r* = 0.323 *p* = 0.124 *r* = 0.084 *p* = 0.695

*Note*: Analyses were performed using Spearman correlation, *p < 0.05.

### 3.6 Change in CS of P300 Amplitude as a Biomarker for Treatment Outcome

**FIGURE 7:**
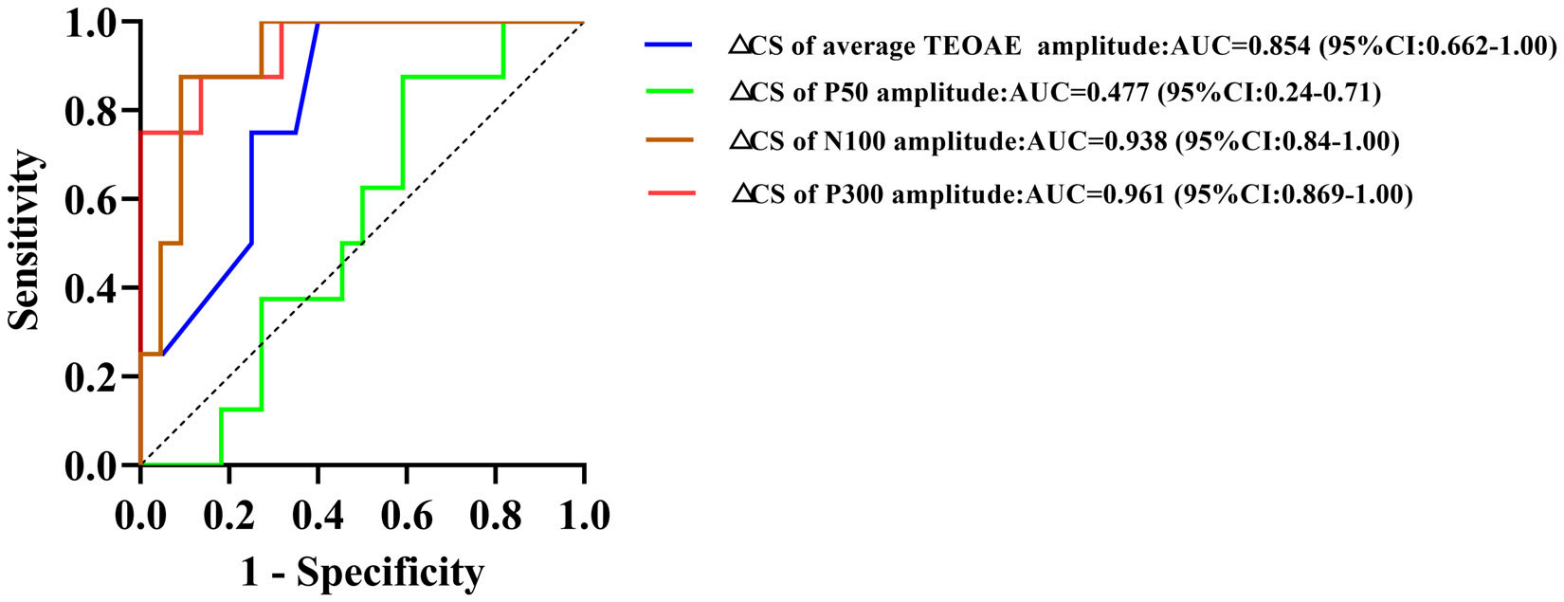
ROC curve distinguishing responders from non‐responders following personalized masking therapy, based on pre‐ to post‐therapy change in contralateral suppression (ΔCS) of CAEP/TEOAE metrics. Curve‐predictor mapping: solid red—ΔCS of P300 amplitude; solid brown—ΔCS of N100 amplitude; solid green—ΔCS of P50 amplitude; solid blue—ΔCS of average TEOAE amplitude. The dashed gray diagonal denotes chance performance. Higher curves indicate better discrimination. Abbreviation: ΔCS, change from baseline.

We evaluated whether △CS of TEOAE amplitude or CAEP amplitude could serve as a biomarker for the outcome of tinnitus masking treatment. The ROC curve analysis revealed that △CS of P300 amplitude had the highest AUC on the ROC curve (AUC = 0.961; 95% CI: 0.888–1.00), indicating high diagnostic accuracy (see Figure 7).

## 4 Discussion

This study demonstrated that chronic tinnitus is associated with a significant reduction in CS of CAEP, specifically in the N100 and P300 components. These findings provide strong evidence that tinnitus involves impaired central auditory inhibition. Furthermore, following 3 months of sound masking therapy, the patients who responded to treatment showed notable improvements in the CS of N100 and P300 amplitudes, paralleling reductions in tinnitus severity. This suggests that at least a portion of the central inhibitory dysfunction in tinnitus is reversible with appropriate therapy.

### 4.1 Contralateral Suppression: Anatomical and Physiological Mechanisms

The MOC system is a key efferent pathway that mediates CS in the auditory system. MOC neurons originate in the superior olivary complex and project to the cochlea, where they synapse on outer hair cells (OHC). Activation of the MOC reflex by sound (especially noise in the opposite ear) inhibits OHC motility and reduces the gain of the cochlear amplifier (Yılmaz et al. 2021). This cochlear suppression is objectively evidenced by a decrease in otoacoustic emission amplitude during contralateral acoustic stimulation (Schochat et al. 2012). By damping OHC‐driven cochlear gain, the MOC efferent system effectively improves the auditory signal‐to‐noise ratio and helps the ear filter out background noise (Yılmaz et al. 2021). Such peripheral inhibition is thought to protect the inner ear from overstimulation and enhance speech perception in noisy environments.

CS is not limited to the cochlea; its effects propagate to higher auditory centers. Schochat et al. (2012) demonstrated that introducing white noise to the opposite ear led to prolonged response latencies and reduced amplitudes in both early brainstem waves and later cortical potentials. In their data, the N1 component (≈100 ms) was significantly attenuated by contralateral noise, reflecting reduced cortical excitation, and the P300 wave showed a notable increase in latency under noise conditions. These findings indicate that the auditory efferent reflex arc extends beyond the periphery, exerting top‐down inhibitory modulation on neural processing at multiple levels. Consistent with this, a recent study in school‐age children by Ubiali et al. (2025) found that contralateral white noise caused significant delays in early cortical responses (prolonging P1 latency) and in higher‐level processing (prolonging P300 latency), while specifically reducing the amplitude of the N1 wave during speech‐evoked potentials (Ubiali et al. 2025). This convergence of evidence confirms that CS engages a multi‐level network of inhibitory influences, from the brainstem MOC reflex up to cortical auditory processing.

This multi‐tiered suppression mechanism is critical for maintaining stable auditory perception and has important clinical implications. By dynamically regulating cochlear gain and cortical responsiveness, the efferent system helps prevent sensory overload and improves the focus on relevant sounds in complex acoustic environments. Deficits in these inhibitory pathways have been linked to auditory processing disorders and to the pathophysiology of tinnitus. Rauschecker et al. (2015) specifically proposed that a frontostriatal “gatekeeper” circuit (ventromedial prefrontal cortex and nucleus accumbens) filters auditory information via descending pathways, and that compromising this top‐down gate can lead to persistent phantom percepts such as tinnitus (Rauschecker et al. 2015). Moreover, understanding these central gating mechanisms may provide new impetus for tinnitus treatment. Thus, CS and its underlying efferent mechanisms not only contribute to robust listening in noise but also offer a framework for exploring interventions in disorders of auditory inhibition.

### 4.2 Impaired Contralateral Suppression in Tinnitus: Central Inhibitory Dysfunction

CS of CAEP is markedly blunted in tinnitus. In our controls, broadband noise to the opposite ear reduced N100 and P300 amplitudes as expected; in tinnitus listeners, CS was negligible or, for N100, sometimes reversed, indicating a failure of central inhibitory gating.

Animal and human data converge on this interpretation. Noise‐exposed rats that develop behavioral tinnitus show down‐regulated GABAergic currents and receptors in the inferior colliculus and auditory cortex, producing hyperexcitability (Wang et al. 2011). Electro‐ and magnetoencephalography (MEG) in patients reveals decreased alpha and elevated gamma power—hallmarks of cortical disinhibition—and impaired paired‐click sensory gating (Campbell et al. 2019; De Ridder et al. 2015; Schoisswohl et al. 2021). Young adults with normal thresholds but chronic tinnitus suppress the second P2 far less than controls, and source analysis implicates weakened frontal inhibitory networks (Campbell et al. 2018). Enhanced onset‐offset CAEP reported by Morse and Vander Werff likewise points to the auditory cortex operating at abnormally high gain (Morse and Vander Werff 2023). Our observation that contralateral noise fails to attenuate N1/P3 thus fits a wider pattern of defective central gating.

Peripheral measures echo the cortical findings. Although baseline TEOAEs were normal, tinnitus ears exhibited significantly weaker CS at 2 kHz and for broadband averages. Reduced MOC reflex strength has also been documented in noise‐induced tinnitus (Lalaki et al. 2011). Together, these results suggest a pan‐auditory inhibitory deficit extending from the brainstem efferent to the cortex.

Shore et al. (2016) proposed that loss of cochlear input triggers “maladaptive plasticity,” combining hyperactivity with weakened inhibition across the auditory hierarchy. The present CS data provide direct human evidence for this model: when the efferent system and cortical interneurons cannot downregulate gain, spontaneous or irrelevant activity is unmasked and perceived as tinnitus. Restoring inhibition—whether through sound therapy, neuromodulation, or pharmacologic potentiation of GABA—therefore remains a central therapeutic goal.

### 4.3 Restoration of Inhibitory Function With Tinnitus Masking Therapy

A key result of the present study is that 3‐month, individualized masking therapy partially reinstated central inhibition in tinnitus. Post‐therapy, N100 and P300 amplitudes recorded without contralateral noise increased—consistent with greater cortical responsiveness once the phantom percept receded—while their contralateral‐suppression (CS) magnitudes also rose. Although whole‐group CS changes did not reach significance, the effective subgroup showed significantly larger CS gains than non‐responders, and ΔCS for both N100 and P300 correlated with ΔTHI (*r* ≈0.64). Hence, the degree to which masking normalized inhibitory gating predicted symptomatic relief, supporting the view that restoring central inhibition is a principal mechanism of benefit.

These findings echo earlier work showing that tinnitus masking therapy reduces tinnitus‐related hyperactivity, presumably by re‐engaging inhibitory networks (Hobson et al. 2012; Neff et al. 2019; Schad et al. 2018; Chen et al. 2021; Lv et al. 2021). Longitudinal MRI confirms such plasticity: Chen et al. (2021) documented normalization of aberrant auditory‐limbic connectivity and microstructural recovery in frontal and cerebellar pathways after 6 months of sound therapy, while Lv et al. (2021) reported strengthened auditory resting‐state networks. Our EEG data provide complementary, time‐resolved evidence that cortical gating itself can improve within a few months.

Peripheral measures mirrored the cortical changes. Absolute TEOAE amplitudes were unchanged, but CS of TEOAEs at 1–3 kHz increased significantly, implying a more effective MOC reflex. Masking may therefore “train” the efferent system—via sustained, low‐level stimulation—to recalibrate central gain and strengthen descending inhibition. Similar improvements in inhibitory neurotransmission have been shown in animal models exposed to enriched acoustic environments (Cheng et al. 2023; Sturm et al. 2017; Zhu et al. 2014). In sum, adult central auditory inhibition remains plastic: targeted acoustic stimulation can enhance both cortical and efferent suppressive mechanisms, and the extent of CS restoration tracks clinical improvement. These results endorse sound‐based approaches as a low‐risk avenue for rebalancing excitation–inhibition dynamics in chronic tinnitus

### 4.4 △CS of P300 Amplitude Served as a Biomarker for Tinnitus Treatment

The significant differences in the CS of CAEP amplitude between tinnitus patients and healthy controls, especially in the P300 component, indicate that CS could serve as a useful biomarker for tinnitus diagnosis. Our ROC curve analysis confirmed the diagnostic utility of P300 CS amplitude, with an AUC of 0.776, suggesting fair diagnostic accuracy. This is in line with findings from recent studies that have investigated the use of auditory evoked potentials as biomarkers for tinnitus (Cardon et al. 2020; Cardon et al. 2022; Mannarelli et al. 2017; Morse and Vander Werff 2023; Sedley et al. 2019). For instance, Cardon et al. (2022) developed a model for detecting tinnitus based on CAEP, brain signal variability, and cognitive performance, demonstrating that tinnitus patients exhibit reduced P300 responses, decreased activity in key brain regions (e.g., temporal cortex, parahippocampus, and insula), and increased brain signal variability. This model achieved an accuracy of 75% and an area under the ROC curve of 0.86, highlighting the potential value of combining these biomarkers in tinnitus detection and emphasizing the role of top–down information processing in tinnitus perception.

More importantly, our study highlights that the change in CS (△CS) of P300 amplitude could serve as a reliable biomarker for assessing tinnitus treatment outcomes. The significant improvement in CS of P300 amplitude following tinnitus masking therapy underscores its potential to reflect changes in central inhibition and auditory processing. This enhancement in P300 amplitude correlates with the clinical reduction in tinnitus severity, as measured by THI scores, supporting the hypothesis that restoring central inhibition through tinnitus masking therapy can alleviate tinnitus symptoms. An event related to attention and cognitive processing is sensitive to alterations in neural excitability and inhibition. The high AUC (0.961) for △CS of P300 amplitude in predicting treatment response further establishes its potential as an effective tool for assessing individual outcomes in tinnitus therapy. These findings are consistent with a recent meta‐analysis (Cardon et al. 2020), which suggests that electrophysiological measures, such as the P300, may serve as valuable tools for monitoring the effectiveness of tinnitus interventions. The ability to differentiate between effective and ineffective treatments using neurophysiological markers like △CS of P300 amplitude represents a significant advancement in clinical practice. This approach could allow for more personalized and targeted interventions, improving treatment outcomes for tinnitus patients.

In sum, contralateral‐suppressed P300 is poised to become a practical, objective tool for diagnosing tinnitus, forecasting treatment response and tailoring individualized therapeutic pathways.

### 4.5 Limitations and Future Directions

Despite promising findings, this study has several limitations. First, our cohort was intentionally restricted to normal‐hearing tinnitus participants to isolate central inhibitory mechanisms while minimizing peripheral confounds. This design strengthens internal validity but limits generalizability to the broader tinnitus population with hearing loss; future work should include such cohorts to test external validity. Second, the sample size was modest and the study was conceived as a mechanism‐focused exploratory investigation; accordingly, no a priori power analysis was undertaken. While several key effects reached statistical significance, non‐significant results—particularly within‐subject changes in CS after therapy—should be interpreted cautiously due to potential type II error. Third, our follow‐up captured short‐term outcomes after a 3‐month masking therapy; the durability of CS changes and their relationship to sustained symptom relief require longer‐term observation in larger, prospectively powered, and preferably multi‐center studies.

This study primarily focused on central auditory cortex processing. Future research should also investigate other non‐auditory brain networks, such as the limbic system, as dysfunction in these areas could contribute to tinnitus perception. Moreover, the role of other neurophysiological biomarkers, such as auditory brainstem responses or MEG, should be explored to gain a more comprehensive understanding of the cortical mechanisms involved in tinnitus and its treatment. Combining these approaches with functional neuroimaging techniques will deepen our understanding of tinnitus‐related neural plasticity and help develop more personalized treatment strategies.

## 5 Conclusion

CS of CAEP emerges from our study as both a window into tinnitus pathophysiology and a potential clinical tool. Anatomically and functionally, it reflects the integrity of the MOC efferent loop and cortical inhibitory networks—systems that are crucial for normal auditory filtering and are disrupted in tinnitus. The reductions in N100 and P300 suppression we observed in tinnitus patients reinforce the view that tinnitus is characterized by central inhibitory insufficiency or “neural noise filtering” failure. Importantly, the partial reversal of these abnormalities with sound therapy offers hope that the tinnitus brain is not irreversibly locked in a maladaptive state; rather, its inhibitory circuits can be retuned with appropriate stimulation. For clinicians and researchers, incorporating CS measures into auditory evoked potential testing could improve tinnitus assessment by adding objective neurophysiological criteria. Further studies in larger populations and with diverse tinnitus interventions will be valuable to confirm the robustness of CS in CAEP as a biomarker. If corroborated, this approach could pave the way for more personalized tinnitus management, where treatments are guided and evaluated through the lens of each patient's unique neurophysiological profile. Ultimately, by restoring the balance between excitation and inhibition in the auditory system—as evidenced by normalized CS—we move closer to breaking the cycle of tinnitus and improving the quality of life for those affected.

## Author Contributions

**Zhou Qian**: conceptualization, investigation, methodology, formal analysis, writing–original draft, writing–review and editing. **Wang Qixuan**: methodology, writing–original draft, writing–review and editing. **Jiang Wenling**: methodology, writing–original draft, writing–review and editing. **Wang Yiting**: investigation, methodology. **Li Haifeng**: investigation, methodology. **Huang Meiping**: investigation, methodology. **Yang Lu**: investigation, methodology. **Ren Yan**: investigation, methodology. **Sheng Haibin**: investigation, methodology. **Li Bei**: investigation, methodology. **Huang Zhiwu**: conceptualization, writing–review and editing, supervision, project administration, funding acquisition.

## Conflicts of Interest

The authors declare no conflicts of interest.

## Ethics Statement

All participants provided written informed consent, and this study was approved by the Ethics Committee of the Shanghai Ninth People's Hospital, located in Shanghai, China (Approval No.: SH9H‐2022‐T379‐1).

## Peer Review

The peer review history for this article is available at https://publons.com/publon/10.1002/brb3.71007

## Data Availability

The data supporting this study's findings are not available to the public due to participants’ confidentiality but are available from the corresponding authors on request.
